# Magnetoelastic Coupled Wave Diffraction and Dynamic Stress Intensity Factor in Graded Piezomagnetic Composites with a Cylindrical Aperture

**DOI:** 10.3390/ma13030669

**Published:** 2020-02-03

**Authors:** Yinhuan Jiang, Chuanping Zhou, Ban Wang, Liqun Wu

**Affiliations:** 1 Jiangsu College of Safety Technology, Xuzhou 221000, China; 2 School of Mechatronic Engineering, China University of Mining and Technology, Xuzhou 221116, China; 3 Key Laboratory of Mechanical Equipment and Technology for Marine Machinery, School of Mechanical Engineering, Hangzhou Dianzi University, Hangzhou 310018, China; 4 College of Electrical Engineering, Zhejiang University, Hangzhou 310027, China

**Keywords:** exponential graded piezomagnetic composites, magnetoelastic coupling wave, aperture, dynamic stress intensity factor

## Abstract

A theoretical method is developed to study the magnetoelastic coupled wave and dynamic stress intensity around a cylindrical aperture in exponential graded piezomagnetic materials. By employing the decoupling technique, the coupled magnetoelastic governing equations are decomposed. Then the analytic solutions of elastic wave fields and magnetic fields are presented by using the wave function expansion method. By satisfying the boundary conditions of the aperture, the mode coefficients, and the analytic solutions of dynamic stress intensity factors are determined. The numerical examples of the dynamic stress intensity factor near the aperture are presented. The numerical results indicate that the incident wave number, the piezomagnetic properties, and the nonhomogeneous parameter of materials highly influence the dynamic stress around the aperture.

## 1. Introduction

Functionally graded material is a kind of composite material whose composition and structure change continuously with gradient. By continuously changing the composition and structure of component materials, the interface disappears, and the properties of the materials change slowly with the composition and structure of those [1,2]. The concept of functionally graded materials can be extended to the piezoelectric materials and piezomagnetic materials to increase the reliability of materials and structures. For functionally graded materials, the most important factor in determining the reliability of those is the graded function. When the elastic modulus and piezomagnetic constant change exponentially, it is easier to realize in the manufacturing process. Therefore, it is of great significance to study the exponentially graded piezomagnetic materials (EGPMs). Furthermore, EGPMs have many unique properties, such as enhanced magneto-mechanical coupling, low power dissipation, and sensitive response, etc. These advantages make them strong candidates for applications as piezomagnetic sensors, magnetic field probes, generators, actuators, resonators, and detectors in the magneto-mechanical systems.

In the progress of connecting, tailoring, and serving of EGPMs, it is inevitable to make openings, and some failures such as cracks and apertures also occur inside the materials or structures. To reduce the stress concentration around the discontinuities in EGPMs, it is vital to consider these apertures and openings. Especially under various dynamic loads, at the discontinuous interface, the dynamic stress concentration may increase markedly, which results in structural strength reduction, structural fatigue, or fracture [3]. 

In the past several years, a great number of numerical, experimental and analytic studies on the coupled magneto-electro-elastic wave propagation in piezomagnetic/ piezoelectric materials have been carried out to improve the strength and stability of piezomagnetic materials and structures [4,5,6,7,8]. Jiao et al. [4] presented a theoretical solution by using the transfer matrix method to study the elastic wave diffraction in a gradient block sandwiched by the piezomagnetic and the piezoelectric half-spaces. Liang et al. [5] studied the magneto-elastic coupling effect in an infinite soft ferromagnetic material with a crack. The magneto-elastic coupling interface problem in soft ferromagnetic materials with cracks subjected to remote uniform magnetic induction was studied [6]. In the light of the functionally composite materials with a crack possessing coupled piezomagnetic, piezoelectric, and magnetoelectric effects, Wang and Mai [7] provided a theoretical method to compute the intensity factors, magnetic inductions and electric displacements near the crack tip. Cao et al. [8] studied the propagation characteristics of Lamb waves in a functionally graded piezoelectric/piezomagnetic composite plate with continuous variation along the thickness direction. The dispersion equation under different boundary conditions is given. The influence of the variation of the parameters on the dispersion curve and the cut-off frequency in the electromagnetic field is discussed in detail. Singh et al. [9] studied the propagation characteristics of the SH waves in two semi-infinite voltage magnetic materials and obtained different forms of the explicit nonuniformity of the dispersion relation. Tian et al. [10] analyzed the propagation of SH wave in the layered structure composed of the functionally graded piezoelectric layer and piezomagnetic half-space. The relationship between the graded coefficient and the thickness of the dielectric layer on SH wave propagation was explained. Zhao et al. [11] studied the propagation of horizontal shear interface waves in functionally graded piezoelectric/functionally graded piezomagnetic bimaterial. The effects of different material combinations, interface imperfections, and material constant graded on phase velocity were illustrated. Pang et al. [12], based on the transfer matrix method and the stiffness matrix method, studied the propagation and location of forward and oblique waves in piezoelectric/piezomagnetic-layered periodic structures. The corresponding characteristics of dispersion curves, localization factors, and response spectra were obtained through calculation. Zhang et al. [13] analyzed the guided waves in functionally graded piezoelectric/piezomagnetic rods with rectangular cross-sections by using the biorthogonal polynomial series method. The corresponding dispersion curves and the distribution of mechanical displacements were obtained through calculation. Kong et al. [14] studied the propagation characteristics of horizontal shear waves in the functional graded piezoelectric bonding layer and the relationship between the inhomogeneity of the functional graded piezoelectric layer and the dispersion behaviors of the SH waves. Sahu et al. [15] studied the propagation characteristics of horizontally polarized shear waves in laminated composite structures sandwiched between the wavy piezomagnetic layer and the elastic substrate through functionally graded piezoelectric materials. The influence of the material graded, the ripple parameters, and the width of the layer under the open circuit and the short circuit of the magnetoelectric power is obtained. 

In this paper, the diffraction and dynamic stress intensity around the aperture in EGPMs under the action of a magneto-elastic coupling wave are studied. The elastic wave fields and magnetic potentials are expanded by using a wave function expansion method. According to the free boundary conditions, the expansion coefficients for the diffraction field in EGPMs are determined. The analytic solution of the dynamic stress intensity factor (DSIF) around the aperture is given. As examples, numerical results of DSIF in EGPMs with an aperture are computed and analyzed with different parameters. The effects of the incident wave number, the piezomagnetic property, and the geometric parameters on DSIFs around the aperture are also analyzed and discussed.

## 2. Dynamics Equation and Maxwell’s Equation in EGPMs and the Solutions

**Figure 1:**
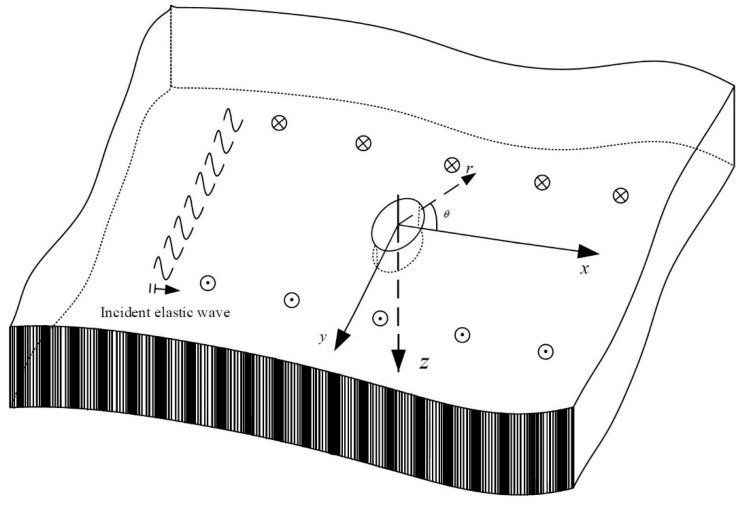
Sketch of SH waves incident in EGPMs with an aperture.

A cylindrical aperture embedded in the infinite EGPM is investigated, as shown in Figure 1. The EGPM is polarized along the *z*-direction with transverse isotropic performance. The material parameters follow the exponential change in the *x*-direction. An SH wave propagates along the positive *x*-direction in the infinite EGPMs. In this case, the anti-plane dynamics equation and Maxwell’s equation in the piezomagnetic materials are:

(1) $$\begin{array}{l} \frac{\partial \tau_{xz}}{\partial x}+\frac{\partial \tau_{yz}}{\partial y}=\rho \frac{\partial^{2}w}{\partial t^{2}}\\ \frac{\partial B_{x}}{\partial x}+\frac{\partial B_{y}}{\partial y}=0 \end{array}$$

where $\tau_{xz},\tau_{yz}$ are the shear stress components, ρ is the density, w is the displacement in the *z*-direction, and $B_{x},B_{y}$ are the magnetic flux densities.

The constitutive relation of EGPMs with magneto-mechanical coupling can be obtained:

(2a) $$\tau_{xz}=h_{15}\frac{\partial \psi }{\partial x}+c_{44}\frac{\partial w}{\partial x},\tau_{yz}=h_{15}\frac{\partial \psi }{\partial y}+c_{44}\frac{\partial w}{\partial y}$$

(2b) $$B_{x}=h_{15}\frac{\partial w}{\partial x}-\mu_{11}\frac{\partial \psi }{\partial x},B_{y}=h_{15}\frac{\partial w}{\partial y}-\mu_{11}\frac{\partial \psi }{\partial y}$$

where $c_{44}$ is the elastic constant of piezomagnetic materials; $h_{15}$ is the piezomagnetic constant of piezomagnetic materials; $\mu_{11}$ is the magnetic permeability; and ψ is the magnetic potential in materials.

Without loss of generality, the steady wave solution is investigated. Set

(3a) $$w=\tilde{w}e^{-i\omega t}$$

(3b) $$\psi =\tilde{\psi }e^{-i\omega t}$$

where ω is the frequency of the incident waves; i is the imaginary unit.

Here we assume that the parameters of EGPMs change continuously by the exponential functions in the *x*-direction

(4) $$c_{44}=c_{440}e^{2\beta x},\rho =\rho_{0}e^{2\beta x},h_{15}=h_{150}e^{2\beta x},\mu_{11}=\mu_{110}e^{2\beta x}$$

where $c_{440},\rho_{0},h_{150},\mu_{110}$ are the initial Young’s modulus, the initial density piezomagnetic constant, and magnetic permeability in vacuum, which are located at the origin of the *x*-axis in the EGPMs, respectively. β is the nonhomogeneous parameter describing the exponent changes along the *x*-direction in the EGPMs.

Substituting Equation (2) into Equation (1), the following expressions are given:

(5a) $$2\beta c_{440}\frac{\partial w}{\partial x}+c_{440}\nabla^{2}w+2\beta h_{150}\frac{\partial \psi }{\partial x}+h_{150}\nabla^{2}\psi =\rho_{0}\frac{\partial^{2}w}{\partial t^{2}}$$

(5b) $$2\beta h_{150}\frac{\partial w}{\partial x}+h_{150}\nabla^{2}w-2\beta \mu_{110}\frac{\partial \psi }{\partial x}-\mu_{110}\nabla^{2}\psi =0$$

where $\nabla^{2}=\frac{\partial^{2}}{\partial x^{2}}+\frac{\partial^{2}}{\partial y^{2}}$ is the Laplace operator.

Assume that a constructor $\varphi =\psi -\frac{h_{150}}{\mu_{110}}w$

(6a) $$\nabla^{2}w+2\beta \frac{\partial w}{\partial x}=\frac{1}{c_{s}^{2}}\frac{\partial^{2}w}{\partial t^{2}}$$

(6b) $$\nabla^{2}\varphi +2\beta \frac{\partial \varphi }{\partial x}=0$$

where $c_{s}^{2}=\sqrt{\chi /\rho_{0}}$ is the propagation velocity of the SH waves, $\chi =c_{440}+h_{150}^{2}/\mu_{110}$.

The steady solution of this problem is studied. Let $w=w_{0}We^{-i\omega t}$, Equation (6) are written as:

(7) $$\nabla^{2}W+2\beta \frac{\partial W}{\partial x}+k^{2}W=0$$

in which ω is the incident wave frequency, and $k=\omega /c_{s}$ is the incident wave number.

We can write the conformational solution of Equation (7) as:

(8) W=exp(−βx)u(x,y)

in which u(x,y) is a conformational function.

Taking Equation (8) into Equation (7), the following expression is derived:

(9) $$\nabla^{2}u+\kappa^{2}u=0$$

where $\kappa =\sqrt{k^{2}-\beta^{2}}$.

Denote $\chi_{1}=\frac{h_{150}}{\mu_{110}}$, we also can derive the magnetic potential form as:

(10) $$\varphi =w_{0}\chi_{1}e^{-\beta x}e^{i(i\beta x-\omega t)}$$

Consider an SH wave propagating along the positive *x*-direction. In the polar coordinate system (*r*, *θ*), the incident waves can be expanded as:

(11a) $$\begin{array}{ll} w^{\left(i\right)} & =w_{0}e^{-\beta x}e^{i\kappa x}\\ & =w_{0}e^{-\beta r\cos\theta }\sum_{n=-\infty }^{\infty }i^{n}J_{n}\left(\kappa r\right)e^{in\theta } \end{array}$$

(11b) $$\begin{array}{ll} \varphi^{\left(i\right)} & =w_{0}\chi_{1}e^{-\beta x}e^{-\beta x}\\ & =w_{0}\chi_{1}e^{-\beta r\cos\theta }\sum_{n=-\infty }^{\infty }i^{n}J_{n}\left(i\beta r\right)e^{in\theta } \end{array}$$

where $\chi_{2}=\frac{h_{150}}{c_{440}},\chi_{3}=\frac{\mu_{110}}{\mu_{0}}$.

According to Equations (6a) and (6b), the scattered field caused by the aperture in EGPMs can be given as:

(12a) $$w^{\left(s\right)}=w_{0}e^{-\beta r\cos\theta }\sum_{n=-\infty }^{\infty }A_{n}H_{n}^{\left(1\right)}\left(\kappa r\right)e^{in\theta }$$

(12b) $$\varphi^{\left(s\right)}=w_{0}\chi_{1}e^{-\beta r\cos\theta }\sum_{n=-\infty }^{\infty }B_{n}H_{n}^{\left(1\right)}\left(i\beta r\right)e^{in\theta }$$

in which $A_{n}$ is the undetermined coefficient to describe the scattered elastic wavefield, $B_{n}$ is the undetermined coefficient to describe the scattered magnetic field, and $H_{n}^{\left(1\right)}\left(\cdot \right)$ is the *n*th order Bessel function of the third kind.

Taking the incident field, scattered field, and reflected fields together, the total field of the elastic waves in the EGPMs is expressed as:

(13a) $$w^{\left(t\right)}=w^{\left(i\right)}+w^{\left(s\right)}$$

The total magnetic potential in the EGPM is expressed as

(13b) $$\psi^{\left(t\right)}=\varphi^{\left(i\right)}+\varphi^{\left(s\right)}+\chi_{1}w^{\left(t\right)}$$

In the aperture, the elastic wave field is non-existent, and the magnetic field is not equal to zero. The magnetic potential in the aperture is expressed as

(14) $$\psi^{c}=w_{0}\chi_{1}e^{-\beta r\cos\theta }\sum_{n=-\infty }^{\infty }C_{n}J_{n}\left(i\beta r\right)e^{in\theta }$$

where $C_{n}$ is the undetermined coefficient to describe the inner magnetic field.

## 3. Boundary Conditions and Determination of Mode Coefficients

In general, due to the existence of aperture, the free boundary condition is studied. We then have the boundary conditions in the form

(15) $$\begin{array}{l} \tau_{rz}|{}_{r=a}=0\\ B_{r}|{}_{r=a}=B_{r}^{c}|{}_{r=a}\\ \psi^{\left(t\right)}|{}_{r=a}=\psi^{c} \end{array}\}$$

According to Equation (15), we can derive the infinite system of linear equations for computing the mode coefficients $A_{n},B_{n},C_{n}$

(16a) $$\begin{array}{l} \left(1+\chi_{1}\chi_{2}\right)\left[i^{n}J_{n}{}^{\prime }\left(\kappa a\right)-\beta \cos\theta i^{n}J_{n}\left(\kappa a\right)\right]\\ +\left(1+\chi_{1}\chi_{2}\right)A_{n}\left[H_{n}^{\left(1\right)}{}^{\prime }\left(\kappa a\right)-\beta \cos\theta H_{n}^{\left(1\right)}\left(\kappa a\right)\right]\\ +\chi_{1}\chi_{2}i^{n}J_{n}{}^{\prime }\left(i\beta a\right)-\chi_{1}\chi_{2}\beta \cos\theta i^{n}J_{n}\left(i\beta a\right)\\ +\chi_{1}\chi_{2}B_{n}H_{n}^{\left(1\right)}{}^{\prime }\left(i\beta a\right)-\chi_{1}\chi_{2}\beta \cos\theta B_{n}H_{n}^{\left(1\right)}\left(i\beta a\right)=0 \end{array}$$

(16b) $$\begin{array}{l} \chi_{3}e^{2\beta a\cos\theta }[i^{n}J_{n}{}^{\prime }\left(i\beta a\right)-\beta \cos\theta i^{n}J_{n}\left(i\beta a\right)\\ +B_{n}H_{n}^{\left(1\right)}{}^{\prime }\left(i\beta a\right)-B_{n}\beta \cos\theta H_{n}^{\left(1\right)}\left(i\beta a\right)]\\ =C_{n}J_{n}{}^{\prime }\left(i\beta a\right)-\beta \cos\theta C_{n}J_{n}\left(i\beta a\right) \end{array}$$

(16c) $$i^{n}J_{n}\left(\kappa a\right)+A_{n}H_{n}^{\left(1\right)}\left(\kappa a\right){+i}^{n}J_{n}\left(i\beta a\right)+B_{n}H_{n}^{\left(1\right)}\left(i\beta a\right)-C_{n}J_{n}\left(i\beta a\right)=0$$

where n=−∞~+∞.

## 4. Dynamic Stress Intensity Factors

According to the definition of the DSIF [16], the DSIF is the ratio of the hoop shear stress around the aperture and the maximum stress [16]

(17) $$DSIF=\frac{\tau_{\theta z}}{\tau_{0}}$$

where $\tau_{0}=w_{0}\chi k$ and

(18) $$\begin{array}{ll} \tau_{\theta z} & =c_{44}\frac{1}{r}\frac{\partial w^{\left(t\right)}}{\partial \theta }+h_{15}\frac{1}{r}\frac{\partial \psi^{\left(t\right)}}{\partial \theta }\\ & =\left(c_{440}+\frac{h_{150}^{2}}{\mu_{110}}\right)w_{0}e^{\beta r\cos\theta }\frac{1}{r}\sum_{n=-\infty }^{\infty }\left(in+\beta r\sin\theta \right)\left[i^{n}J_{n}\left(\kappa r\right)+A_{n}H_{n}^{\left(1\right)}\left(\kappa r\right)\right]e^{in\theta }\\ & +h_{150}\chi_{1}w_{0}e^{\beta r\cos\theta }\frac{1}{r}\sum_{n=-\infty }^{\infty }\left(in+\beta r\sin\theta \right)\left[i^{n}J_{n}\left(i\beta r\right)+B_{n}H_{n}^{\left(1\right)}\left(i\beta r\right)\right]e^{in\theta } \end{array}$$

Thus, the DSIF around the cylindrical aperture in EGPMs is expressed as

(19) $$\begin{array}{ll} DSIF & =e^{\beta r\cos\theta }\frac{1}{kr}\sum_{n=-\infty }^{\infty }\left(in+\beta r\sin\theta \right)\left[i^{n}J_{n}\left(\kappa r\right)+A_{n}H_{n}^{\left(1\right)}\left(\kappa r\right)\right]e^{in\theta }\\ & +h_{150}\frac{\chi_{1}}{\chi }e^{\beta r\cos\theta }\frac{1}{kr}\sum_{n=-\infty }^{\infty }\left(in+\beta r\sin\theta \right)\left[i^{n}J_{n}\left(i\beta r\right)+B_{n}H_{n}^{\left(1\right)}\left(i\beta r\right)\right]e^{in\theta } \end{array}$$

## 5. Numerical Examples Simulation and Discussion

After obtaining the DSIF expression, we can accurately calculate the distributions of DSIF around the cylindrical aperture in EGPMs. Equation (18) is a convergent infinite series; however, in test calculations it is identified that when the truncations *n*≥15 the calculation results can meet the engineering precision requirement. We choose CoFe_2_O_4_ as the materials of the piezomagnetic phase in the EGPMs. The relative material constants are $\rho_{0}=5.3\times 10^{3}kg\cdot m^{-2},$ $c_{440}=45.3\times 10^{9}N\cdot m^{-2},$ $h_{150}=550N\cdot A^{-1}\cdot m^{-1},$ $\mu_{110}=157\times 10^{-6}N\cdot A^{-2}$. For ease of analysis, the variables in calculations are made dimensionless. We take the aperture radius *a* as the characteristic length. Take the dimensionless incident wave number *ka* = 0.1 ~ 3.0 and take the nonhomogeneous parameter *βa* = −0.50 ~ 0.50.

**Figure 2:**
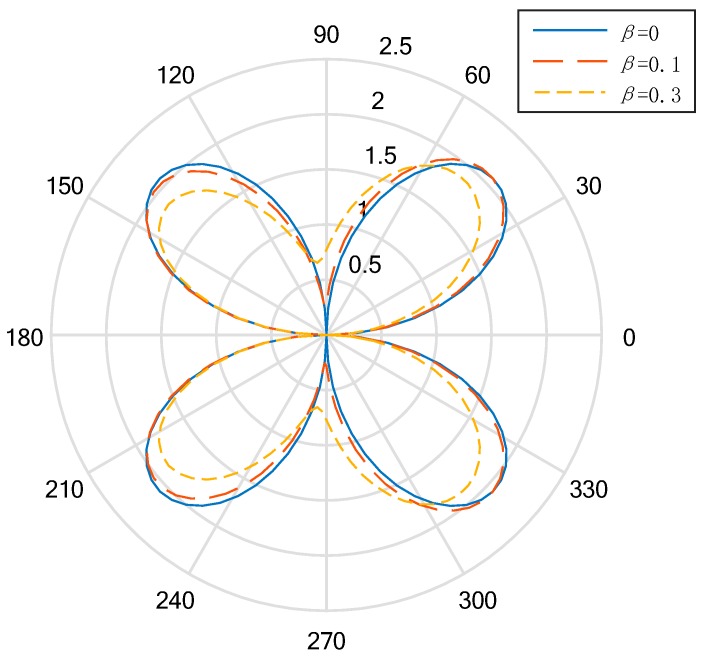
Polar graph of DSIF around the circular aperture (*ka* = 0.5, β≥0).

**Figure 3:**
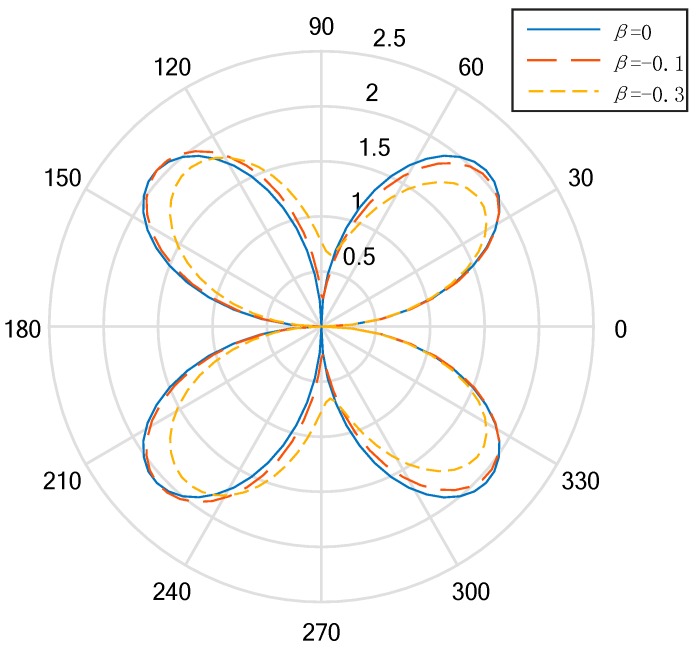
Polar graph of DSIF around the circular aperture (*ka* = 0.5, β≤0).

In the low-frequency condition (*ka* = 0.5), the distribution curve to describe the DSIFs near the cylindrical aperture in the EGPMs is given in Figure 2 and Figure 3. We can see that when the wave number is small, if the nonhomogeneous parameter is βa=0, the DSIFs of the back-wave side (the right half of the figure) and the traveling wave-side (the left half side of the figure) is symmetric about the vertical axis. If the nonhomogeneous parameter is βa≠0, the figures are not symmetric. Also, when the nonhomogeneous parameters |βa| are greater, the asymmetry rates are greater. If the nonhomogeneous parameter are βa>0, the total value of the DSIFs on the back-wave side is larger than the total value of the DSIFs on the traveling wave side. When βa=0.1, the maximum value of the DSIFs of the back side appears near $\theta =\frac{\pi }{4}$ and $\theta =\frac{7\pi }{4}$, and when βa=0.3, the maximum value of the DSIFs of the traveling side appears near $\theta =\frac{7\pi }{9}$ and $\theta =\frac{11\pi }{9}$. With the increase of |βa|, the maximum and minimum of DSIFs shift to the traveling wave, but do not exceed the value of DSIFs when βa=0 near the transverse axis. With the increase of |βa|, the maximum value of the DSIFs decreases and the minimum value of the DSIFs increases.

**Figure 4:**
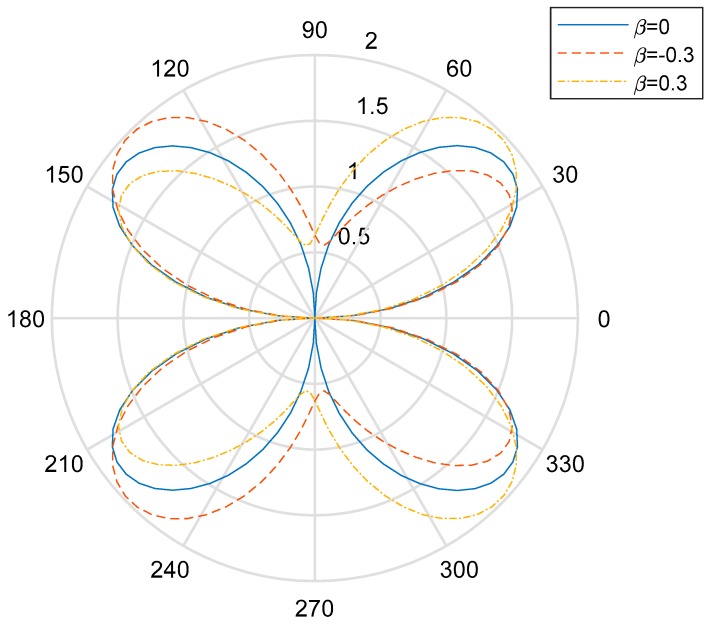
Polar graph of DSIF around the circular aperture with *ka* = 1.0.

**Figure 5:**
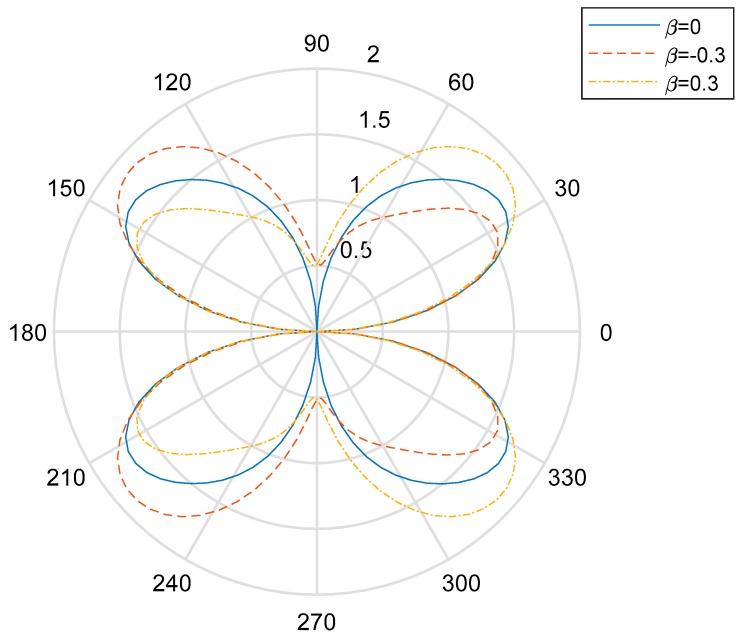
Polar graph of DSIF around the circular aperture with *ka* = 2.0.

**Figure 6:**
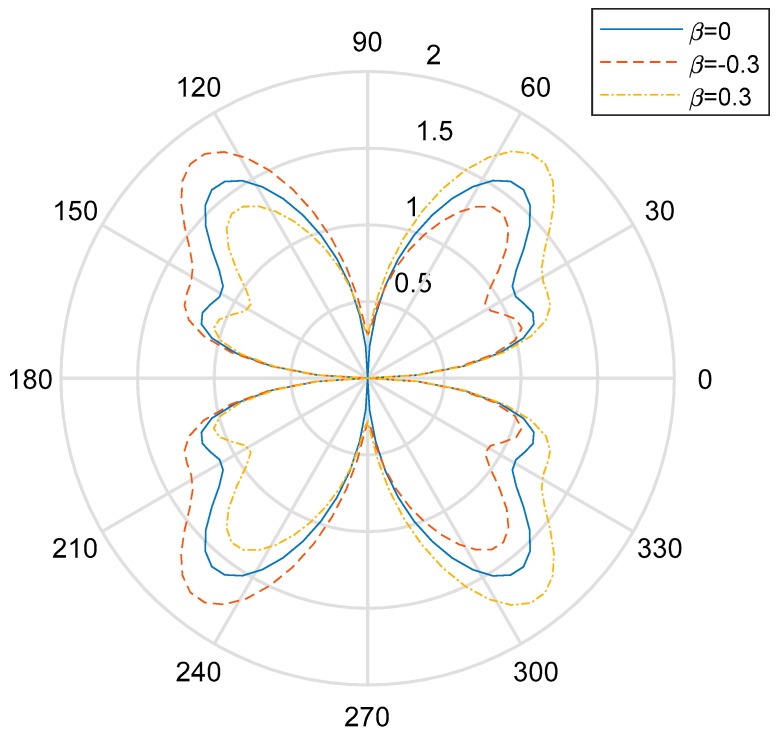
Polar graph of DSIF around the circular aperture with *ka* = 3.0.

**Figure 7:**
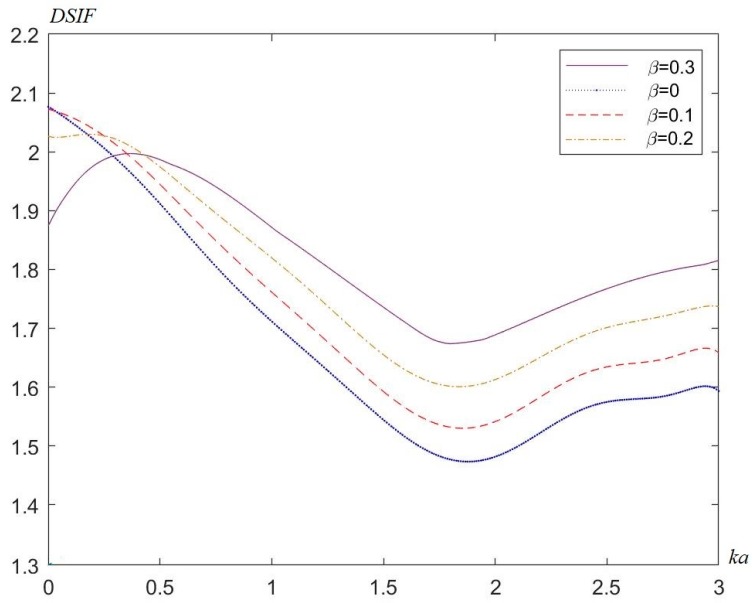
Maximum DSIF around the circular aperture versus the incident wave number *ka*.

Figure 4, Figure 5 and Figure 6 show the distribution of the DSIFs near the cylindrical aperture when the incident wave number is, respectively, ka=1.0,ka=2.0,ka=3.0. The distribution of the value of DSIFs when βa=−0.3 and the distribution of the value of DSIF when βa=3.0 are nearly symmetric with vertical axis. As is shown in Figure 6, when the incident wave number, there are fluctuations in the value of the DSIFs on the back-wave side, and the traveling-wave side. When the nonhomogeneous parameter βa=−0.3, the locations of the minimum value of DSIFs in the fluctuation range is near $\theta =\frac{\pi }{6}$. Figure 7 shows the influence of the incident wave number *ka* with the maximum DSIF around the circular aperture. From Figure 7 we can see that when the maximum DSIF for the homogeneous case is greater than the nonhomogeneous cases. When the nonhomogeneous parameter is greater, the maximum DSIF is smaller.

## 6. Conclusions

In this paper, we investigate the DSIF around a cylindrical aperture in an infinite EGPM under SH waves. The analytical solution and numerical solution of this problem are obtained. From the obtained numerical results, the following conclusions can be drawn:

The wave incident wave numbers highly influence the value and distribution of the DSIFs around the aperture. When the wave incident wave number is great, the DSIF curves show fluctuations. In contrast to the solution in the static case, analyses show that the piezomagnetic properties have a great effect on the dynamic stress in the region of intermediate frequency. Greater incident wave numbers will enhance the piezomagnetic effects on the dynamic stress. Comparing with the homogeneous materials, the nonhomogeneous parameter of EGPMs has a great effect on the value and distribution of the DSIFs around the aperture. The nonhomogeneous parameter can effectively reduce dynamic stress concentration.

The research methods and the numerical results based on this study can be applied in the dynamic analysis and strength design for the structure of EGPMs. And it can provide an important theoretical foundation for the nondestructive evaluation of piezomagnetic materials.
